# Comparative SERS Activity of Homometallic and Bimetallic Core–Satellite Assemblies

**DOI:** 10.3390/nano14181506

**Published:** 2024-09-16

**Authors:** Gianfranco Terrones-Morey, Xiaofei Xiao, Vincenzo Giannini, Alex Fragoso, Luca Guerrini, Nicolas Pazos-Perez

**Affiliations:** 1 Department of Physical and Inorganic Chemistry, Universitat Rovira i Virgili, Carrer de Marcel∙lí Domingo 1, 43007 Tarragona, Spain; gianfranco.terrones@urv.cat; 2 Departament d’Enginyeria Química, Universitat Rovira i Virgili, Països Catalans 26, 43007 Tarragona, Spain; 3 Technology Innovation Institute, Building B04C, Masdar City, Abu Dhabi P.O. Box 9639, United Arab Emirates; xiaofei.xiao@tii.ae (X.X.); vincenzo.giannini@tii.ae (V.G.); 4 Instituto de Estructura de la Materia (IEM-CSIC), Consejo Superior de Investigaciones Científicas, Serrano 121, 28006 Madrid, Spain; 5 Centre of Excellence ENSEMBLE3 sp. z o.o., Wolczynska 133, 01-919 Warsaw, Poland

**Keywords:** plasmonic, nanoparticles, core–satellites, surface-enhanced Raman spectroscopies

## Abstract

The fabrication of core–satellite (CS) assemblies offers a versatile strategy for tailoring the optical properties of plasmonic nanomaterials. In addition to key factors like size, shape, and spatial arrangement of individual components, the combination of plasmonic units with different compositions (e.g., gold and silver) has been demonstrated to produce materials with enhanced properties and functionalities applicable across a range of fields. Notably, several CS assembly variants have emerged as promising substrates for surface-enhanced Raman spectroscopy (SERS). In this study, we address a gap in the knowledge by conducting a systematic cross-comparison of the optical and SERS properties of highly bright homo- and bimetallic CS assemblies. We evaluated the SERS efficiencies of these different superstructures across various excitation wavelengths and supported our findings with numerical simulations. The insights gained from this study offer a valuable foundation for researchers seeking to select and optimize the most suitable CS assemblies for their given SERS application.

## 1. Introduction

The ability to finely tune the properties of plasmonic nanoparticle assemblies is a rapidly evolving field with significant implications across numerous technological applications [1,2,3,4]. The near-field coupling between localized surface plasmon resonances (LSPRs) of individual nanoparticles is fundamental to the appearance of unique optical properties which can be properly tailored through the rational design of plasmonic assemblies composed of metallic units with specific compositions, shapes, and sizes [1,2,5]. Remarkably, the spacing and spatial organization of plasmonic building blocks have dramatic impacts on the near-field enhancements [1,2,4]. As a result, developing methodologies to engineer high-quality nanoparticle assemblies with tunable plasmonic responses, spanning from top-down nanofabrication to bottom-up assembly methods, became one of the most exciting and fast-moving areas in nanoscience research [1,5,6,7,8]. Among others, core–satellite (CS) assemblies, comprising a central core surrounded by smaller peripheral particles, offer a flexible approach to enhancing and controlling optical properties. This class of plasmonic assemblies typically exhibits stable and relatively predictable optical responses while enabling a high degree of material customization [1,4,9].

Surface-enhanced Raman spectroscopy (SERS) benefits from the electromagnetic fields arising from the excitation of LSPRs to intensify the Raman scattering of molecules [10]. However, only those molecules located at or very near the metallic surface can experience a large signal intensification since the near-field intensity of the plasmon-enhanced electromagnetic fields exponentially decreases with distance from the plasmonic material [11]. Notably, the strong coupling of closely spaced plasmonic nanoparticles maximizes the magnitude of such electromagnetic enhancement [12]. As a result, single-molecule SERS detection can be achieved for molecules localized in these narrow interparticle regions (hot spots) [12]. Overall, the ultrasensitivity provided by unique plasmon-mediated enhancement, combined with the intrinsic specificity and experimental flexibility of Raman spectroscopy, has fueled the implementation of SERS in numerous sensing applications, particularly in biological and environmental analyses [13,14,15,16].

CS assemblies that simultaneously meet the criteria of presenting (i) short interparticle gaps within a few nanometers (e.g., <2–3 nm) and (ii) a high number of core–satellite junctions (i.e., large satellite loading) are very promising materials for SERS applications. In this scenario, the assemblies concentrate dense collections of highly efficient hot spots, symmetrically arranged in a small volume. Consequently, they provide high SERS activity as well as minimal variability of the SERS response under different excitation polarization directions [17], thereby enabling the linear optical response necessary for quantitative analysis.

The main synthetic routes for the controlled assembly of plasmonic building blocks rely on top-down lithographic methods on solid supports and bottom-up molecular-assisted assembly of colloidal nanoparticles in suspension [1,9]. Currently, lithographic techniques and bottom-up methods using outstanding molecular tools such as DNA offer a way to finely engineer plasmonic superstructures with well-defined architectures. However, their application to SERS is partially hampered due to the difficulty of reducing interparticle distances to the levels required for large SERS enhancements [1,9]. Thus, since the gap size is primarily determined by the length of the molecular linker, bottom-up approaches using small molecular ligands have been often preferred for generating core–satellite clusters to be used as SERS platforms [18,19,20,21].

Recently [18], we devised a versatile strategy to produce CS plasmonic particles with defined core–satellite gaps (~2–3 nm) and a satellite coverage approaching the jamming limit. These features offer a dense array of isotropically distributed, highly efficient hot-spots, which endow the materials with exceptional optical performance, such as uniform and large SERS enhancement factors [18]. Remarkably, molecular reporters of different natures can be efficiently located at the core–satellite gaps enabling the fabrication of highly bright SERS-encoded particles [22]. Moreover, the proposed method offers a modular design, allowing for the integration of various plasmonic building blocks with minimal adjustments to the general synthesis protocol. Additionally, their relatively small size makes them ideal for quantitative determinations and implementation in size-restricted applications, such as in vivo and in vitro bioimaging [23].

Recent investigations have revealed the distinct advantages of combining compositionally diverse plasmonic units, such as silver (Ag) and gold (Au) [1,24,25,26]. Integrating different materials within a single structure has been demonstrated to produce multicomponent materials with enhanced or novel properties that can be leveraged across various applications, including sensing, energy, and catalysis [1,24,25,26]. Beyond intrinsic plasmonic features, additional factors such as biocompatibility, chemical stability, nanofabrication versatility, and ease of functionalization—factors that typically favor gold-based substrates over silver—are typically considered in the design of a SERS platform for a given application [7]. As of now, the literature lacks systematic studies that provide a comprehensive and organized comparison of the optical and SERS properties of multicomponent CS assemblies. In this work, to address this knowledge gap, we have expanded the compositional diversity of CS assemblies by combining four sets of building blocks: ~16 nm Au or Ag spherical satellites and ~90 nm Au or Ag spherical cores. These components were combined to create both homometallic (Ag@Ag, Au@Au) and bimetallic (Au@Ag, Ag@Au) assemblies. The SERS efficiencies of these different classes of constructs were investigated across various excitation wavelengths and interpreted with the support of numerical calculations. This research provides valuable insights for other researchers, serving as a foundation for selecting and optimizing the most suitable core–satellite assemblies for specific applications.

## 2. Materials and Methods

### 2.1. Materials

Trisodium citrate dihydrate (≥99.5%, C_6_H_5_Na_3_O_7_·2H_2_O), absolute ethanol (≥99.9%, EtOH), L-ascorbic acid (≥99.0%, AA), 4-mercaptobenzoic acid (≥99%, 4MBA), 11-mercaptoundecanoic acid (≥95%, MUA), silver nitrate (≥99.9999%, AgNO_3_), gold(III) chloride trihydrate (≥99.9%, HAuCl_4_·3H_2_O), sodium hydroxide (≥98%, NaOH), tetraethoxysilane (≥99.999%, TEOS), branched polyethylenimine (≥99.5%, PEI), and magnesium sulfate (≥98%, MgSO_4_) were purchased from Thermo Fisher Scientific Inc. All reactants were used without further purification and all glassware was cleaned with aqua regia before the experiments. Milli-Q water (18 MΩ cm^−1^) was used in all aqueous solutions.

### 2.2. Synthesis of Ag Nanoparticle Cores

#### 2.2.1. Synthesis of Citrate-Capped Silver Nanoparticle Seeds of ~50 nm Diameter

Silver nanoparticles (AgNPs) of ~50 nm diameter were produced as follows. In total, 100 mL of H_2_O were heated until boiling and, under strong magnetic stirring, a mixture of ascorbic acid (AA, 100 μL, 0.1 M) and trisodium citrate dihydrate (1.364 mL, 0.1 M) was then added. After 1 min, a second mixture obtained after 5 min incubation of AgNO_3_ (297.6 μL, 0.1 M) and MgSO_4_ (223.6 μL, 0.1 M) solutions was also added. A condenser was used to prevent solvent evaporation. The boiling and stirring continued for 1 h, resulting in nanoparticles with an average size of 48 ± 3 nm.

#### 2.2.2. Synthesis of Citrate-Capped Silver Nanoparticle Cores of ~90 nm Diameter

AgNPs of ~90 nm diameter were synthesized using a modified kinetically controlled seeded growth method [27]. The produced AgNPs of ~50 nm diameter were used as seeds to overgrow them as follows. Immediately after their synthesis, the seed colloidal suspension was cooled down to a constant value of 90 °C. A mixture of AA (18.2 µL, 0.1 M) and trisodium citrate dihydrate (248 µL, 0.1 M) was added to the colloidal solution under vigorous stirring. Then, 2 min later, a AgNO_3_ solution (297.6 µL, 0.1 M) was injected under vigorous stirring, and the stirring continued for 30 min. This process (sequential addition of a mixture of AA-trisodium citrate and AgNO_3_ solution) was repeated 5 times to obtain Ag NPs of ~90 nm diameter. In each step, one aliquot of 1 mL was extracted for further characterization using transmission electron microscopy (TEM) and UV-vis spectroscopy. Finally, the excess of citrate from the colloidal suspension was removed using centrifugation (4200 rpm, 10 min) and redispersion in MilliQ water. The concentration of NPs was approximately the same as the original seed particles (1.3 × 10^10^ NPs/mL, [Ag^0^] = 5.2 × 10^−4^ M). The nanoparticle size was estimated to be 93 ± 10 nm (Figure S1).

### 2.3. Synthesis of Au Nanoparticle Cores

#### 2.3.1. Synthesis of Citrate-Capped Gold Nanoparticle Seeds of ~13 nm Diameter

Small gold nanoparticles (AuNPs) of ~13 nm diameter were synthesized as seeds by adding trisodium citrate dihydrate (500 µL, 2.2 mM) and HAuCl_4_ (833.0 µL, 0.1 M) to a boiling mixture of 100 mL of MilliQ water under vigorous stirring. A condenser was used to prevent the evaporation of the solvent. The boiling and stirring continued for 30 min, yielding spherical AuNPs of ~13 ± 1 nm diameter.

#### 2.3.2. Synthesis of Citrate-Capped Gold Nanoparticle Cores of ~90 nm Diameter

AuNPs of ~90 nm diameter were synthesized using a modified kinetically controlled seeded growth method [28]. The temperature of the boiling colloidal suspension of AuNPs seeds was first decreased to 90 °C. Subsequently, 2 mL of trisodium citrate dihydrate (60 mM) was injected. Then, 2 min later, 2 mL of HAuCl_4_ (25 mM) was also added under strong stirring, and the mixture was allowed to react for 45 min. This process was repeated twice. After that, the sample was diluted by extracting 50 mL of the sample and adding 50 mL of Milli-Q water. This process (sequential dilution and addition of C_6_H_5_Na_3_O_7_·2H_2_O and HAuCl_4_) was repeated 4 times to finally obtain AuNPs of ~90 nm. In each step, one aliquot of 1 mL was extracted for further characterization using TEM and UV-vis spectroscopy. Finally, the NPs were cleaned using centrifugation (4200 rpm, 10 min) to eliminate the excess of citrate and redispersed in MilliQ water to achieve an NPs concentration of 1.6 × 10^10^ NPs/mL, [Au^0^] = 5.2 × 10^−4^ M. The nanoparticle size was estimated to be 87 ± 7 nm (Figure S1).

### 2.4. Synthesis of Au and Ag Nanoparticle Satellites

#### 2.4.1. Synthesis of Citrate-Capped Ag Nanoparticle Satellites

Spherical silver nanoparticles with a diameter of approximately 15 nm were synthesized using a previously described method [29]. Briefly, a mixture containing ascorbic acid (100 µL, 0.1 M) and trisodium citrate dihydrate (600 µL, 0.1 M) was added to 100 mL of boiling Milli-Q water. After 1 min, a solution that was previously incubated for 5 min containing silver nitrate (198.4 µL, 0.1 M) and iron(III) nitrate (79.4 µL, 0.01 M) was injected into the solution. Boiling and stirring were continued for 1 h. The nanoparticle’s diameter was estimated to be ~15 ± 1 nm (Figure S1) with an approximate concentration of 5.7 × 10^12^ NPs/mL and [Ag^0^] = 3.5 × 10^−4^ M.

#### 2.4.2. Synthesis of Citrate-Capped Au Nanoparticle Satellites

Spherical gold nanoparticles with an approximate diameter of 16 nm were synthesized using a modification of the well-known Turkevich method [30]. In brief, 833 µL of an aqueous solution of HAuCl_4_ (0.1 M) was added to a boiling solution of trisodium citrate dihydrate (500 µL, 2.2 mM) under vigorous stirring. The nanoparticle size was estimated to be 16 ± 1 nm (Figure S1), with an estimated concentration of ~7.6 × 10^13^ NPs/mL and [Au^0^] = 5.3 × 10^−4^ M.

### 2.5. Ag and Au Cores Codification and PEI Wrapping

#### 2.5.1. Ag and Au Cores Codification

To codify the produced Ag and Au cores, a previous stabilization with MUA (1.25 molecules of MUA/nm^2^) was performed [31]. To do this, a solution containing MUA (7.4 μL, 10^−3^ M in EtOH) was rapidly added to 20 mL of Au and Ag cores (~90 nm, [Ag^0^] = 2.7 × 10^−4^ M, [Au^0^] = 2.5 × 10^−4^ M, in Milli-Q water) under vigorous stirring. Subsequently, 100 µL of NaOH solution (0.23 M, in Mili-Q water) was added. The mixture was left undisturbed overnight. The codification process was then performed by adding the Raman label (RL) 4-MBA (177.8 μL, 10^−4^ M in EtOH) at a ratio of 3 molecules of RL/nm^2^, and then was left undisturbed for 5 days. After codification, NPs were cleaned using centrifugation (4200 rpm, 10 min) and redispersed in Milli-Q water to achieve a [Ag^0^] of 5.0 × 10^−4^ M and a [Au^0^] of 5.0 × 10^−4^ M.

#### 2.5.2. PEI Wrapping of Ag and Au Cores

Both codified Ag and Au NPs cores were then wrapped with a positive polyelectrolyte monolayer by adding, drop by drop, 2 mL of the NPs to an aqueous PEI solution (2 mL, 2 g L^−1^, previously sonicated for 30 min) under vigorous stirring, and then were left undisturbed overnight [18]. After that, the excess of PEI was cleaned using centrifugation (4000 rpm, 8 min) and redispersed in Milli-Q water. This process was repeated 5 times to ensure the complete removal of unbonded PEI.

### 2.6. Formation of Core–Satellite Assemblies

CS assemblies were generated using a slight adaptation of a previously published method [18]. In total, 2 mL of either PEI-coated 4-MBA labeled Ag core ([Ag^0^] = 3.2 × 10^−4^ M, 7.8 × 10^9^ NPs/mL) or PEI-coated 4-MBA labeled Au core ([Au^0^] = 2.5 × 10^−4^ M, 7.7 × 10^9^ NPs/mL) suspensions were added dropwise to separate suspensions containing 2 mL of Ag or Au satellites ([Ag] = 2.5 × 10^−4^ M and [Au] = 2.5 × 10^−4^ M) under vigorous stirring. The core–satellite ratio was maintained at ~1:250. The mixtures were stirred overnight using an orbital mixer.

### 2.7. Silica Encapsulation and Purification

Silica encapsulation of CS was carried out using a modified Stöber method [32]. The final concentrations of H_2_O, EtOH, and NaOH were adjusted to 7.94 M, 14.60 M, and 0.4 mM, respectively, thereby yielding a EtOH/H_2_O molar ratio of 1.84. Next, 9.6 μL of TEOS (10% *v*/*v* in EtOH) was added for each 1 mL of H_2_O. The solution was energetically shaken and left undisturbed at room temperature for 14 h. Finally, purification was proceeded by first removing excess reactants by removing the supernatants after centrifugation (3 × 3000 rpm for 6 min). The pellets were then redispersed in 1 mL of Milli-Q water, and silica-coated CS assemblies were separated from the residual small, unbound silica-coated satellites via sedimentation. This involved allowing the solution to stand undisturbed for 96 h in a 1.5 mL conical centrifuge tube, followed by the removal of the supernatant. This process was repeated twice.

### 2.8. Core–Satellite Samples for SERS Analysis

SERS experiments were performed on CS colloidal suspensions with a cluster concentration of ~3.9 × 10^9^ CS/mL, which corresponds to a final concentration of 4-MBA of ~5 × 10^−7^ M. The laser was focused on the colloidal suspension using a macrolens (0.5 NA, 15 mm working distance). The excitation of the samples was carried out with 514, 633, and 785 nm laser lines.

### 2.9. Instrumentation

The morphology of individual nanomaterials (size, shape, and distribution) was investigated using Transmission Electron Microscopy (TEM, JEOL USA, Inc., Peabody, MA, USA); electron micrographs were recorded with JEOL JEM-1011 operating at 80 kV. On the other hand, the optical properties, specifically the localized surface plasmon resonances (LSPRs), of the nanoparticles in suspension were probed via UV-Visible spectroscopy, which also indirectly informed us about the particle size and aggregation; UV–vis spectra were recorded using an Aligent Varian Cary 5000 spectrophotometer (HITACHI, Tokyo, Japan). Silver and gold concentration for NPs were calculated using the Lambert–Beer law using an extinction coefficient of 1.42 × 10^11^ M^−1^ cm^−1^ for Ag and 5.17 × 10^10^ M^−1^ cm^−1^ for Au [33]. SERS spectra were collected in backscattering geometry with a Renishaw Invia Reflex system equipped with a 2D-CCD detector and a Leica confocal microscope. The spectrograph used a high-resolution grating (1200 g cm^−1^) with additional bandpass filter optics.

### 2.10. Numerical Simulations

The numerical simulations were carried out using a three-dimensional finite-difference time-domain (3D FDTD, Ansys/Lumerical) method. To simplify the simulation, the satellite spheres were only arranged on a plane in the center of the core sphere [18]. The incidence light propagated to the negative z-axis with a linear x-polarization. Perfectly matched layers (PML) were used in all directions to absorb incident light with minimal reflections. A two-dimensional monitor was used to extract the electric field on a plane in the center of the core sphere. The mesh was set to 0.4 nm to resolve the fine features. In this calculation, the permittivity of gold and silver were adapted to the datasets of [34,35]. The background was assumed to be water with a refractive index of 1.33.

## 3. Results

The CS assembly building blocks consist of citrate-capped spherical-like silver or gold colloids, which were synthesized in two different sizes: large ~90 nm diameter cores and small ~16 nm diameter satellites (Figure S2). Both Ag and Au cores were prepared using kinetically controlled seeded growth methods [27,28]. After the synthesis, an additional centrifugation and redispersion step was carried out to remove the excess citrate from the medium and yield colloidal suspensions with approximately equal nanoparticle concentrations. These plasmonic units were then combined to form both homometallic (Ag@Ag, Au@Au) and bimetallic (Au@Ag, Ag@Au) clusters using a slightly modified version of a previously described bottom-up electrostatically mediated assembly protocol [18].

**Figure 1:**
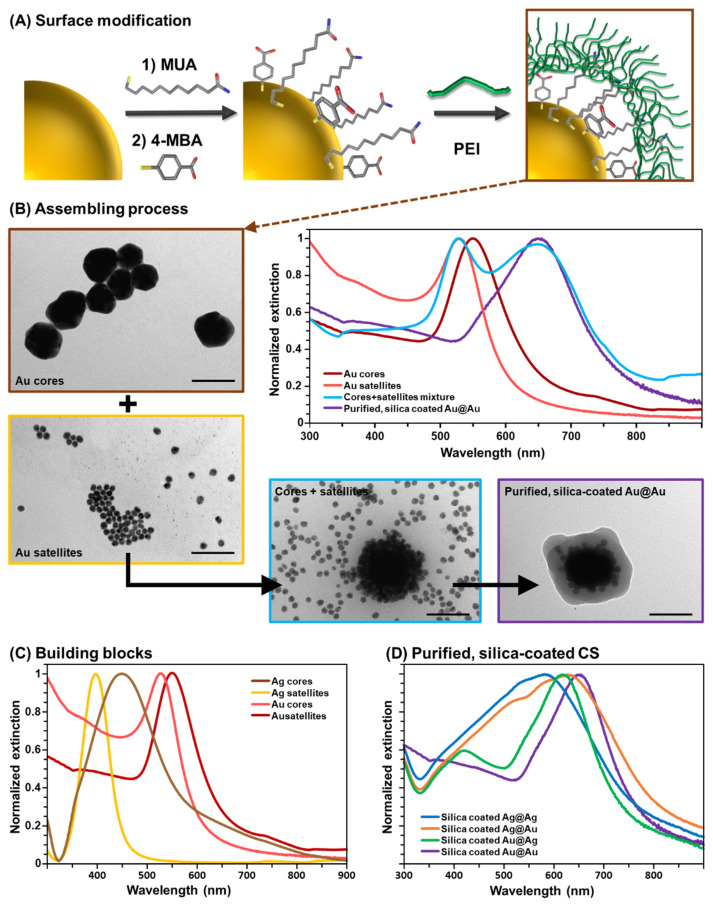
(**A**) Scheme of the surface modification of the plasmonic cores with MUA, 4-MBA, and PEI. (**B**) Outline of the assembling process of Au@Au. Representative TEM images of Au cores, Au satellites, the mixture of cores and satellites (redispersed pellet after overnight sedimentation), and purified silica-coated Au@Au CS. Normalized extinction spectra of the corresponding suspensions. (**C**) Normalized extinction spectra of the plasmonic building blocks. (**D**) Normalized extinction spectra of the purified silica-coated CS assemblies.

Briefly, a mixed monolayer of a stabilizing agent (mercaptoundecanoic acid, MUA) and a SERS-active molecule (4-mercaptobenzoic acid, 4-MBA) was generated at the core surfaces via covalent attachment of MUA and 4-MBA mercapto groups to the metallic surfaces (Figure 1A) [31]. These negatively charged cores were then wrapped with a layer of branched polyethylenimine (PEI), a positively charged polymer. The resulting charge inversion allowed for the subsequent electrostatic adhesion of the citrate-capped negatively charged satellites (Figure 1A). The satellites were added in large excess to maximize their loading onto the nanoparticle cores while minimizing the potential aggregation of poorly coated core particles.

As displayed by the representative TEM images shown in Figure 1B, which illustrate the assembling process of homometallic gold particles, the mixture of cores and satellites will eventually result in the combination of unbound satellites and CS architectures. The assemblies feature a dense isotropic distribution of small particles covering the larger inner cores. On the other hand, in the corresponding extinction spectra, the optical contributions of the CS clusters are barely distinguishable because of the large excess of unbound satellites in suspension [18]. To this end, we carried out an overnight gravity sedimentation of the original mixture, followed by the removal of the supernatant, before acquiring the extinction spectrum of the redispersed pellet. This allowed us to appreciate two main features in the extinction spectra (Figure 1B, blue curve): one centered at ~520 nm, assigned to residual unbound Au satellites, and one at ~650 nm, mainly ascribed to diverse plasmon coupling interactions arising from core–satellite and satellite–satellite proximity [18,36]. In the case of core–satellite junctions, the range of interparticle distances is primarily restricted by the overall molecular size of the MUA/PEI double-layer, which has been estimated to fall within the ~2–3 nm range (Figure S3) [18]. On the other hand, the uncontrolled satellite–satellite aggregation at the core surfaces results in a wide-ranging gap separation, which, in turn, leads to a broader distribution of plasmonic resonances [18,36]. Nonetheless, due to the much larger optical cross-sections of the 90 nm core particles, the overall plasmonic response of the CS assembly remains largely determined by the plasmonic behavior of the inner core [18,36,37].

However, achieving high-yield isolation of the CS fraction without compromising cluster stability requires more complex strategies than simple sedimentation of the original mixture. Such approaches often involve imparting sufficient colloidal stability to the assemblies before proceeding with purification steps [38]. For instance, it has been shown that direct silica encapsulation of all particles in the core–satellite mixture provides a simple approach to facilitate the subsequent efficient separation of the lighter unbound silica-coated satellites from the silica-coated assemblies through post-centrifugation and sedimentation steps [18]. Indeed, the extinction spectrum of the silica-coated Au@Au clusters after such purification does not exhibit the characteristic plasmonic contribution from unbound Au satellites at shorter wavelengths (Figure 1B, purple curve), thus revealing the true optical profile of the assemblies. Similar observations can be made for the extinction spectra of all remaining colloidal building blocks and purified silica-coated CS assemblies (Figure 1C,D, respectively).

As compared to individual Au building blocks, Ag colloids exhibit LSPR maxima at shorter wavelengths (~397 and ~450 nm for satellites and cores, respectively). Accordingly, the silica-coated CS assemblies yield extinction spectra with progressively red-shifted maxima as their composition transitions from pure silver to pure gold (~582 nm for Ag@Ag, ~617 nm for Au@Ag, ~627 nm for Ag@Au, and ~650 nm for Au@Au). In the case of CS assemblies comprising Ag cores, the plasmonic profile is much broader than those equipped with Au cores. Since the plasmonic response of the clusters is primarily determined by the plasmonic behavior of the large inner particle, we can attribute this difference to the significant damping of the plasmonic resonances in gold that emerges below ~600 nm [11].

**Figure 2:**
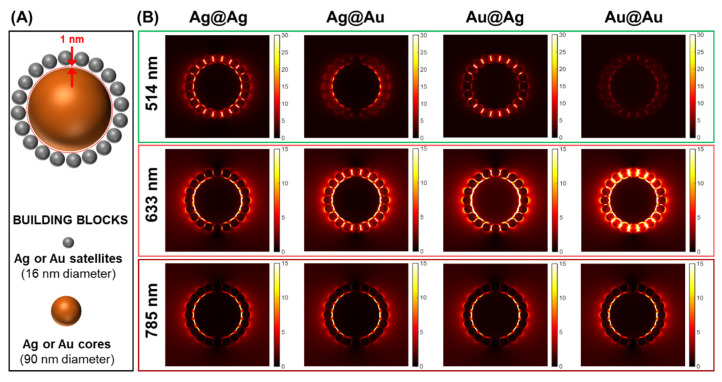
(**A**) Schematic representation of the modeled system, where the core–satellite interparticle gap is maintained at 2 nm. (**B**) Electromagnetic near-field intensity calculations illustrate the spatial near-field map of $\left|E\right|/\left|E_{0}\right|$ under various excitation wavelengths (514, 633 and 785 nm). The value of $\left|E\right|/\left|E_{0}\right|$ was calculated at the location marked by the red dotted circle in (**A**), which intersects the centers of the core–satellite gaps, situated 1 nm from the metallic surfaces.

To acquire a better understanding of the near-field coupling arising from the nanoparticle assembly in CS clusters and the impact on the intensification of the Raman scattering from the SERS probe, numerical simulations of the distribution of the field enhancement intensity |*E*|/|*E*_0_| were carried out using a three-dimensional finite-difference time-domain (3D FDTD, Ansys/Lumerical) method [18]. Recognizing the difficulty of performing numerical simulations on a system analogous to the experimental assemblies (i.e., inner core surrounded by a dense collection of satellites approaching the jamming limit), we investigated a simplified model. This approach aimed to provide valuable insights into the qualitative changes in the relative SERS response of the various CS structures. This model system consists of a 90 nm sphere surrounded by 20 equally spaced 16 nm diameter satellites, which were arranged on a plane that crosses the center of the core (Figure 2A, see experimental section for additional information), and immersed in water (n = 1.33). The field enhancement was calculated at the center of the 2 nm core–satellite gap (i.e., at 1 nm distance from the metallic surface) to yield stable values. The wavelength dependence of the electric field distribution was studied at three different wavelengths (514, 633 and 785 nm), which are those typically used in SERS experiments. Figure 2B shows the corresponding maps for all four classes of assemblies, while Figures S4–S6 also display the field enhancement intensity calculated for the individual building blocks as well as for the satellite corona. As seen in the FDTD simulations, large EM field intensities are localized at the core–satellite junctions, providing an abundant number of efficient hotspots evenly arranged around the central particle.

SERS analysis of the CS assemblies in suspension is performed using a macrolens. This yields a straightforward, robust, and quantitative characterization of their SERS response, since it represents a highly averaged signal from a large number of clusters in continuous Brownian motion within the scattering volume of the objective. However, post-treatments (i.e., silica coating and purification steps) of the original core + satellite mixtures only enable the recovery of an unknown fraction of the original CS due to various experimental factors such as incomplete recovery via centrifugation, adhesion to tube walls, incomplete separation in the sedimentation steps, etc. To circumvent this issue, we directly investigated the cores and satellites mixtures upon the electrostatic assembly and before the silica coating and post-purification steps. Indeed, the SERS signals arising from these mixtures are uniquely related to the CS clusters equipped with 4-MBA molecules covalently attached to the core surface, while the large excess of unbound satellites remains “SERS silent”.

**Figure 3:**
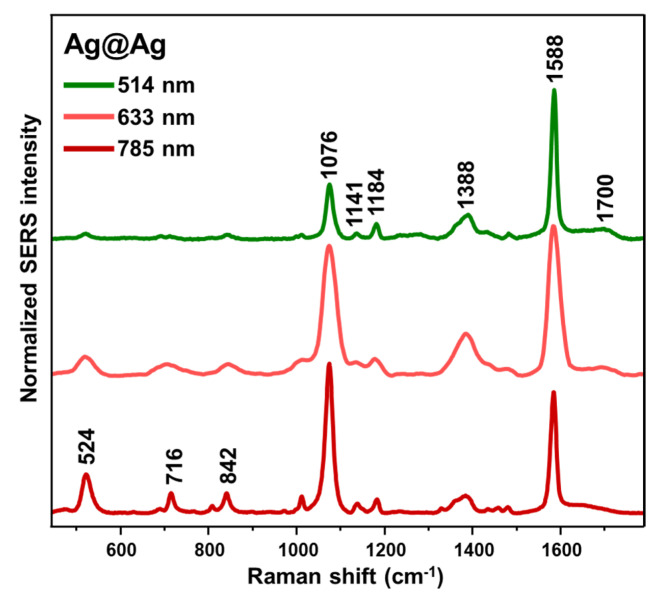
Normalized SERS spectra of 4-MBA on Ag@Ag assemblies at different excitation wavelengths.

SERS spectra of 4-MBA for all four mixtures exhibit a high degree of similarity across each excitation wavelength (Figure S7). On the other hand, we do observe a significant increase in the relative intensity of the bands at shorter Raman shifts as the excitation laser is moved to higher wavelengths (see, for example, Figure 3 for Ag@Ag assemblies). As 4-MBA does not undergo resonance Raman scattering with the investigated lasers, this behavior can be solely attributed to the uneven quantum efficiency of the CCD detector for different excitation sources [39]. The intense bands at ~524, 1076 and 1588 cm^–1^ have been ascribed to C=C stretching, ring breathing, and out-of-plane ring deformation modes, respectively [40,41]. On the other hand, the broad feature approximately centered at ~1390 cm^−1^ is the result of diverse contributions of COO^–^ symmetric stretching vibrations, which are extremely sensitive to alterations in local pH [42] and to the complexation of various metal ions [43].

In the absence of significant alterations to the Raman polarizability tensor of the probe chemisorbed onto the metallic surface, the overall SERS amplification is dominated by EM enhancement contribution [11]. In assemblies of plasmonic nanostructures, such as CS clusters, the extent of EM enhancement largely depends on the specific features of the nanomaterials, including their composition, size, shape, geometric arrangement, and interparticle distance [11]. These features collectively determine the quality and position of the red-shifted LSPRs that arise from plasmon coupling at the interparticle gaps (hot spots).

When the wavelength of the excitation laser matches the resonance conditions of these gap plasmon resonances, extremely intense electromagnetic fields are localized in these regions, resulting in SERS enhancement factors (EFs) that are several orders of magnitude larger than those of individual nanoparticles. For the smallest EFs, values can reach as high as 10^10^–10^11^ [11,44]. Therefore, in core–satellite assemblies characterized by short interparticle gaps, the overall SERS signal is expected to be dominated by the fraction of probe molecules that are spatially localized in these regions. In the case of our four classes of investigated CS assemblies, all structural features that influence the degree of field enhancement at the core–satellite gaps were kept constant, except for the composition of the building blocks. This makes the composition the key factor for interpreting variations in the observed SERS intensities. In this context, as previously mentioned, the use of gold is unsuitable for SERS applications with a green laser, unlike silver, due to the relatively high value of the imaginary part of the refractive index below ~600 nm [11].

**Figure 4:**
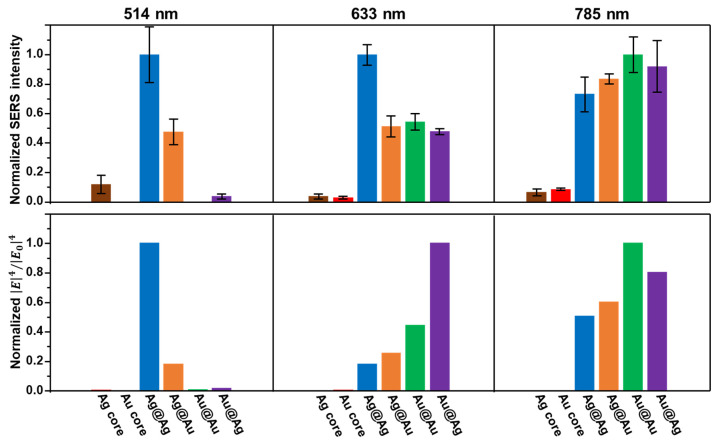
Peak height of the C=C stretching band at ~1588 cm^−1^ (top graphs, N = 3) and calculated fourth power of the local electric field enhancement ${\left|E\right|}^{4}/{\left|E_{0}\right|}^{4}$ (bottom graphs) for plasmonic cores and CS assemblies. In both cases, values were normalized for each excitation wavelength.

The peak height of the ring breathing mode was selected to quantitatively monitor the SERS response of the CS assemblies. The corresponding normalized values for each excitation wavelength are displayed in Figure 4. The experimental results are also compared to the theoretical SERS enhancement factors (EFs) at the center of the core–satellite gap, assuming a direct proportionality of EFs to the fourth power of the local electric field enhancement (${\left|E\right|}^{4}/{\left|E_{0}\right|}^{4}$) [45]. Except for the case of Ag@Ag assemblies at 633 nm, the experimental results qualitatively match the relative distribution of the EFs at the different core–satellite gaps. As expected, detectable SERS signals under the 514 nm laser were only recorded for colloidal suspensions containing Ag cores, with the largest values provided by the homometallic Ag@Ag assemblies, followed by Ag@Au clusters and individual Ag cores. Conversely, at the longest excitation wavelength (785 nm), the optical absorption of gold closely approaches that of silver, making all four classes of assemblies highly effective SERS substrates. However, CS equipped with gold cores do appear to sustain moderately larger EM enhancements than assemblies with silver cores. This difference, given that all of the other structural parameters are equal, can be possibly attributed to the gap plasmon resonance maxima of gold-core CS clusters being closer to the excitation wavelength. This finding is further supported by the results of the numerical simulations.

Regardless of the excitation wavelength, the experimental results consistently exhibit larger SERS intensities from individual cores than those suggested by the theoretical calculations. A likely explanation is related to the uncontrolled formation of small aggregates consisting of closely spaced cores to an extent that is not detectable in the extinction spectra, but it is sufficient to result in an appreciable intensification of the overall SERS signal, which is mostly ascribed to that small fraction of 4-MBA molecules located at the core-core hot spots. Finally, unlike for 514 nm and 785 nm, the 633 nm excitation lies in the spectral range where steeper changes in the plasmonic response occur (Figure 1D). Consequently, it is expected that the structural differences between the architecture of the real CS assemblies (Figure 1B) and the model system (Figure 2A) could lead to significant discrepancies between the experimental and simulated results, which did not otherwise apply to the other excitation wavelengths. Nonetheless, these experimental results collectively show an improvement in the relative SERS performance of gold-based materials when the excitation wavelength shifts from the green laser to longer wavelengths. This trend becomes more pronounced at 785 nm, which is consistent with the strengthening of the LSPRs for gold.

## 4. Conclusions

In summary, this study provides a systematic cross-comparison of the optical and SERS properties of geometrically equivalent but compositionally diverse core–satellite (CS) assemblies. Homometallic and bimetallic CS were synthesized using an electrostatically mediated assembly approach, which enabled us to combining four sets of building blocks (~16 nm Au or Ag spherical satellites and ~90 nm Au or Ag spherical cores) into assemblies characterized by a dense collection of peripheral particles closely attached to the inner core. The assembly process was monitored through TEM and UV-Vis spectroscopy. The SERS response of the different CS assemblies was assessed at three common excitation wavelengths (514, 633 and 785 nm) using 4-mercaptobenzoic acid as a non-resonant molecular probe. Numerical simulations of field enhancement intensity in the core–satellite gaps were also conducted to validate the findings. Overall, the experimental data highlight that the intensity of the SERS signal in such CS is primarily determined by the composition of the inner core. Specifically, we observed a significant increase in the relative SERS efficiency of gold core-based assemblies compared to silver core assemblies as the excitation wavelength shifted from the visible (green) region to the near-infrared (NIR), where Au@Au clusters even outperformed the Ag-based assemblies. The insights from this study offer a valuable foundation for researchers aiming to select and optimize the most suitable CS assemblies for specific SERS applications.

## Data Availability

The raw data supporting the conclusions of this article will be made available by the authors on request.

## Supplementary Materials

The following supporting information can be downloaded at: https://www.mdpi.com/article/10.3390/nano14181506/s1, Figure S1: Size-distribution histograms of Au and Ag individual nanoparticles. Figure S2: Representative TEM images of colloidal Au and Ag cores and satellites and their corresponding silica-coated CS assemblies. Figure S3: Representative TEM images of Au@Au assemblies. Figure S4: Simulation of the electric field maps for individual building blocks, CS assemblies, and satellite coronas (λ = 514 nm). Figure S5: Simulation of the electric field maps for individual building blocks, CS assemblies, and satellite coronas (λ = 633 nm). Figure S6: Simulation of the electric field maps for individual building blocks, CS assemblies, and satellite coronas (λ = 785 nm). Figure S7: Normalized SERS spectra of 4-MBA labeled CS assemblies at different excitation wavelengths.
