# Supplementary material for: Comparative SERS Activity of Homometallic and Bimetallic Core–Satellite Assemblies
Source: Nanomaterials (Basel). 2024 Sep 16;14(18):1506. doi: 10.3390/nano14181506 (PMC11435184; doi:10.3390/nano14181506)
Supplement: Supplementary file 1 [file nanomaterials-14-01506-s001.zip › nanomaterials-3158446-supplementary.pdf]

nanomaterials

Supplementary Materials

Comparative SERS Activity of Homometallic and Bimetallic Core–Satellite Assemblies

Gianfranco Terrones-Morey [1,2], Xiaofei Xiao [3], Vincenzo Giannini [3,4,5], Alex Fragoso [2,*], Luca Guerrini [1,*] and Nicolas Pazos-Perez [1,*]

[1] Department of Physical and Inorganic Chemistry, Universitat Rovira i Virgili, Carrer de Marcel·lí Domingo 1, 43007 Tarragona, Spain; gianfranco.terrones@urv.cat
[2] Departament d'Enginyeria Química, Universitat Rovira i Virgili, Països Catalans 26, 43007 Tarragona, Spain
[3] Technology Innovation Institute, P.O. Box 9639, Building B04C, Masdar City, Abu Dhabi, United Arab Emirates; xiaofei.xiao@tii.ae (X.X.), vincenzo.giannini@tii.ae (V.G.)
[4] Instituto de Estructura de la Materia (IEM-CSIC), Consejo Superior de Investigaciones Científicas, Serrano 121, 28006 Madrid, Spain
[5] Centre of Excellence ENSEMBLE3 sp. z o.o., Wolczynska 133, 01-919 Warsaw, Poland
* Correspondence: nicolas.pazos@urv.cat (N.P.-P); luca.guerrini@urv.cat (L.G.); alex.fragoso@urv.cat (A.F.)

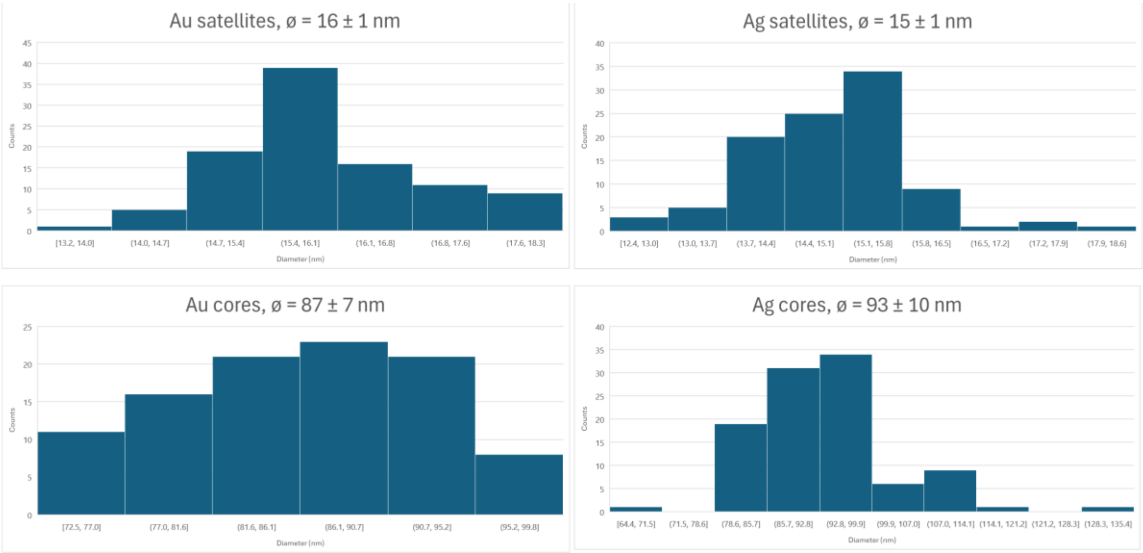

Figure S1. Size-distribution histograms of Au and Ag individual nanoparticles.

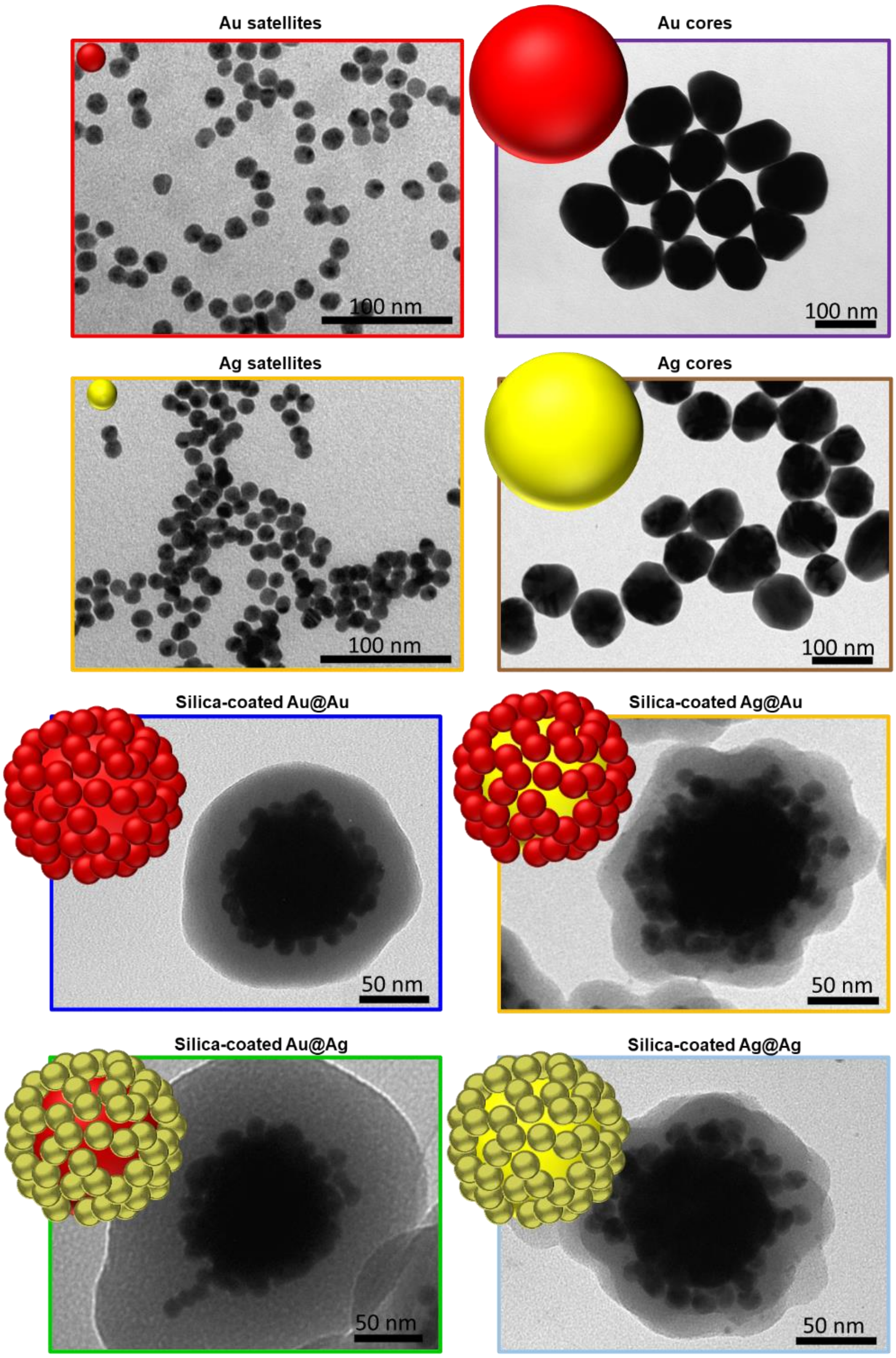

Figure S2. Representative TEM images of colloidal Au and Ag cores and satellites and their corresponding silica-coated CS assemblies.

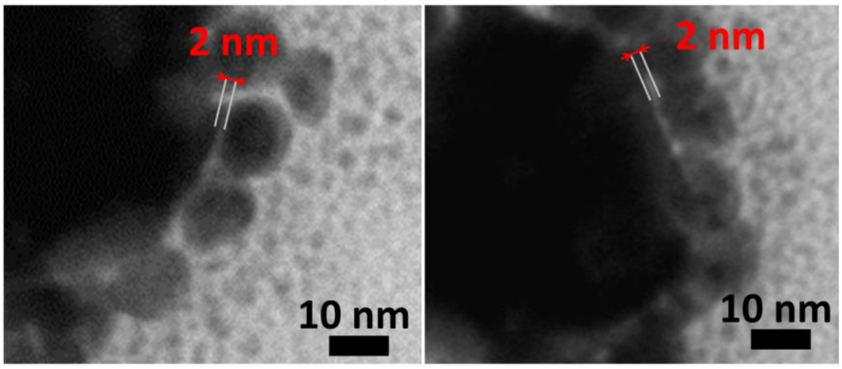

Figure S3. Representative TEM images of Au@Au assemblies.

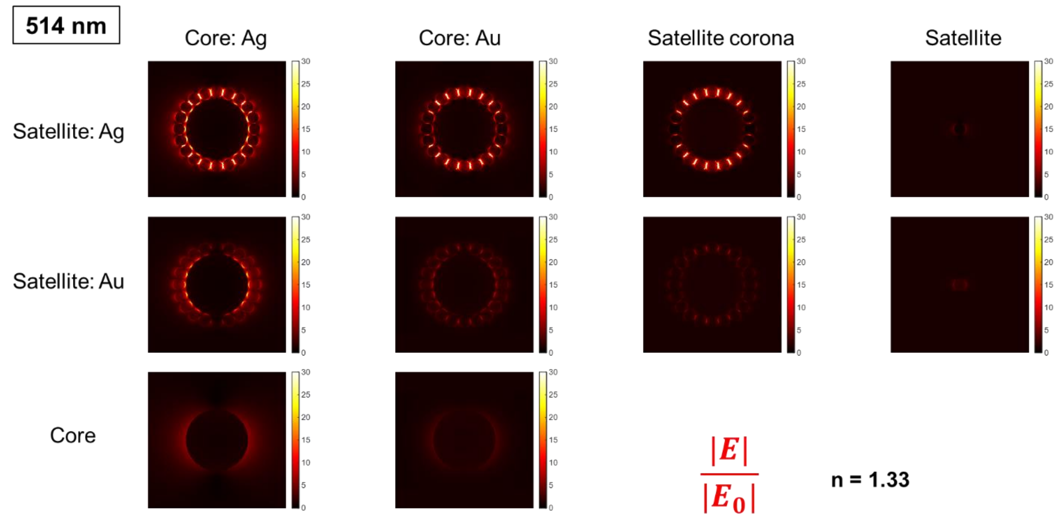

Figure S4. Simulation of the electric field maps for individual building blocks, CS assemblies, and satellite coronas (λ = 514 nm).

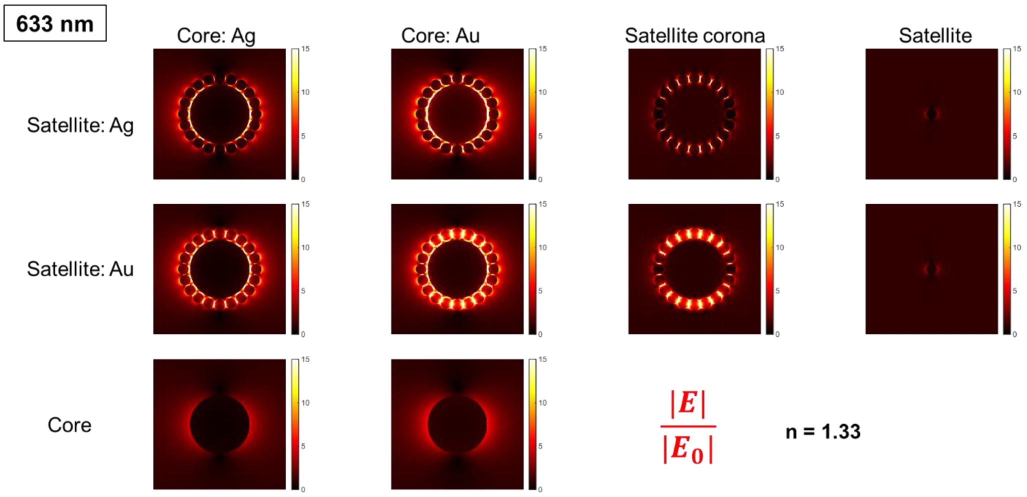

Figure S5. Simulation of the electric field maps for individual building blocks, CS assemblies, and satellite coronas (λ = 633 nm).

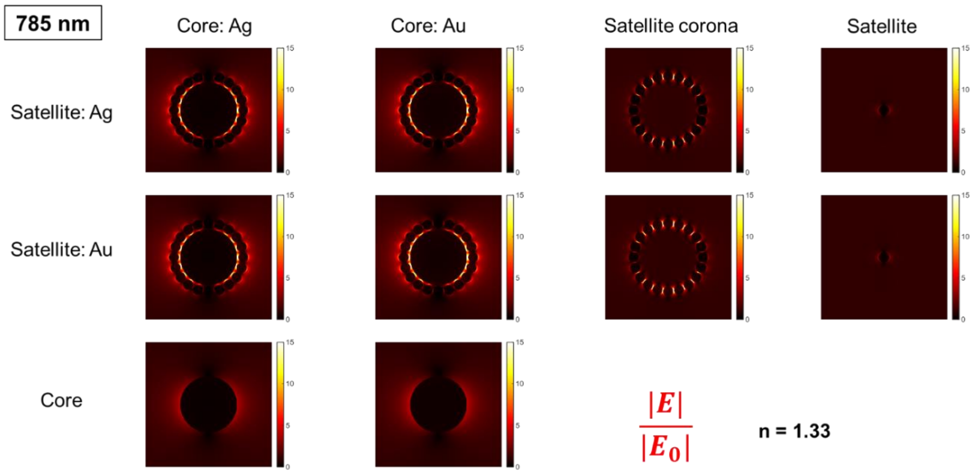

Figure S6. Simulation of the electric field maps for individual building blocks, CS assemblies, and satellite coronas (λ = 785 nm).

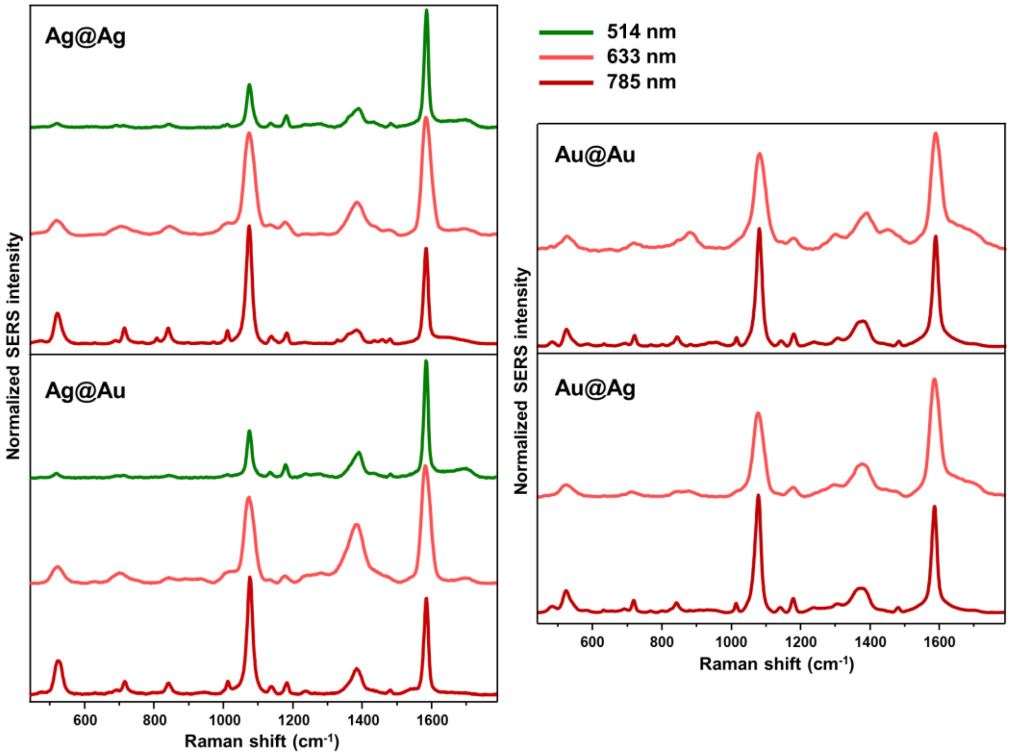

Figure S7. Normalized SERS spectra of 4-MBA labeled CS assemblies at different excitation wavelengths.
